# How leader–member exchange affects university teachers’ scholarship of teaching and learning performance: a comprehensive analysis of psychological capital, LMX differentiation, and traditionality

**DOI:** 10.3389/fpsyg.2025.1514700

**Published:** 2025-04-28

**Authors:** Jiashuai Fang, Qian Zhang, Peiya Rao, Guanghua Ouyang

**Affiliations:** ^1^School of Education Science, Xinyang Normal University, Xinyang, China; ^2^Faculty of Education, Central China Normal University, Wuhan, China

**Keywords:** university teachers, grass-roots teaching organization, leader-member exchange, scholarship of teaching and learning (SoTL) performance, psychological capital, leader-member exchange differentiation

## Abstract

**Introduction:**

The growing focus on teaching research in higher education due to rapid socio-economic development has diminished the importance of teaching. The Scholarship of Teaching and Learning (SoTL) addresses this by promoting systematic research on teaching practices. However, integrating SoTL into organizational performance management remains challenging, especially in China. This study examines how leader-member exchange (LMX) affects university teachers’ SoTL performance, considering psychological capital as a mediator and LMX differentiation and traditionality as moderators.

**Methods:**

Based on social exchange theory, resource conservation theory, and social comparison theory, this study developed a cross-level three-order regulatory moderated mediation model. Data were collected from 351 university teachers across 68 grassroots teaching organizations in three universities in central China. Measures included LMX, psychological capital, traditionality, and SoTL performance. The analysis involved descriptive statistics, correlation analysis, and PROCESS macro for mediation and moderation effects.

**Results:**

The results showed a strong positive correlation between LMX and SoTL performance (*r* = 0.742) and between psychological capital and SoTL performance (*r* = 0.677). Psychological capital partially mediated the relationship between LMX and SoTL performance, accounting for 26.866% of the total effect. The study also found a significant three-order interaction effect of LMX differentiation and traditionality on the relationship between LMX and psychological capital. Specifically, the positive impact of LMX on psychological capital was most pronounced when LMX differentiation was high and teacher traditionality was low.

**Discussion:**

This study highlights the critical role of LMX in enhancing university teachers’ SoTL performance through psychological capital. It underscores the importance of psychological capital as a mediator and the complex moderating effects of LMX differentiation and traditionality. The findings suggest that optimizing LMX relationships, developing psychological capital, and leveraging traditionality can significantly improve SoTL performance. The study provides new insights into the application of LMX theory in higher education and offers practical strategies for enhancing teaching quality in Chinese universities.

## 1 Introduction

As the social economy grows, a rapid scientific and technological progress demands colleges and universities to prioritize scientific research on teaching. This trend has exacerbated the longstanding tension between teaching and research, gradually marginalizing the importance of teaching. However, Ernest Boyer’s multidimensional view of academia offers a theoretical framework to address this imbalance. Boyer posits that academic activities should encompass four dimensions: discovery, integration, application, and teaching. Among these dimensions, effective teaching shares the same characteristics as other forms of academic research and constitutes an independent academic activity (Boyer, 1990). For undertaking teaching research, Shulman (2001) introduced the concept of the scholarship of teaching and learning (SoTL), emphasizing systematic research by teachers on their own teaching practices and students’ learning, and disseminating the results through academic standards. While SoTL is widely recognized in theory, its practical implementation still faces significant challenges and therefore poor (Vikhnevich, 2025), particularly in integrating individual teaching practices with organizational performance management and in discussing the mechanisms underlying its impact on academic performance. This gap is particularly evident in the field of higher education in China, where research on SoTL performance remains scarce.

In China, many teaching and research activities are conducted by grassroots teaching organizations (You, 2012). These organizations, which are the fundamental units within colleges and universities, are directly responsible for teaching activities. They may include teaching and research sections, curriculum groups, teaching teams, and other forms. Consisting of one or two principals and several team members, these grassroots organizations are responsible for organizing teaching tasks, disseminating teaching policies, and facilitating the exchange of teaching experiences. They play critical roles in educational reform and talent development within colleges and universities (Lu and Zhang, 2018). Despite their significant roles in China’s higher education system, the interpersonal dynamics within these organizations, particularly the relationship between teachers and leaders, have not been thoroughly examined yet. In the context of Chinese higher education, concept of “guanxi” (“connections”) is deeply embedded in organizational culture and significantly influences personal career development and the achievement of organizational goals (Yang, 2005). Leader–member exchange (LMX) theory offers a new perspective on how these relationships influence academic performance by examining the quality of interactions between leaders and members (Gerstner and Day, 1997). While LMX theory has been widely applied in organizational settings, studies also suggest that leader–member exchange within schools can enhance teachers’ job performance (Turgut and Tokmak, 2015; Tekin, 2018). However, in the field of higher education, the impact of LMX on SoTL performance and the underlying mechanisms remain poorly understood. Only a few studies have explored this topic.

This study aims to address this theoretical gap by investigating the impact of LMX on the SoTL performance of university teachers. Specifically, this study explores the mediating role of psychological capital and the moderating effects of LMX differentiation and traditionality. Psychological capital is a core competency and psychological resource that can stimulate positive organizational behavior, with a proven significant impact on job performance across various contexts (Zhang et al., 2023). LMX differentiation refers to the effects of leader–member interactions at the organizational level, which may be either positive or negative depending on specific circumstances (Matta and Van Dyne, 2020). Traditionality, deeply rooted in the Confucian culture, significantly influences role formation and expression in the interactions between individuals and leaders (Hu et al., 2020). This study constructs a third-order moderated mediation model, incorporating psychological capital as a mediating variable and examining the moderating effects of LMX differentiation and traditionality.

In summary, the contributions of this study are three-fold. First, this study extends the application of LMX theory to higher education, highlighting its unique role in the Chinese academic environment and contributing to the study of organizational behavior in colleges and universities. Second, this study also explores the mediating role of psychological capital, enriching our understanding of how LMX affects SoTL performance. Third, this study further examines whether LMX differentiation at the organizational level and traditionality at the individual level moderate the effect of LMX, expanding our understanding of its role in varying contexts.

## 2 Theoretical background and research hypotheses

### 2.1 Leader–member exchange of university teachers and SoTL performance

Role orientation and role identity, which are fundamental internal drivers of university teachers’ professional development, play critical roles in organizational management. Within the grassroots teaching organizations at universities, the responsibilities of teachers and organizational leaders overlap significantly, with both roles embodying a blend of moral, social, and academic dimensions (Chen, 2013). Furthermore, organizational leaders, who typically possess extensive teaching experience and professional development resources, are responsible for uniting the professional team, overseeing teaching tasks, and enhancing teaching quality. Previous studies have already indicated that the development of college teachers’ role identity is influenced by a variety of factors. This developmental process, resulting from teachers’ interaction and integration of external environmental resources, is shaped by their own knowledge structures, ideologies, and behavioral patterns (Agila, 2023). In this process, knowledge and information shared among the team members is crucial (Carmeli et al., 2013). In particular, LMX, as a key mechanism for knowledge and information exchange between university teachers and organizational leaders, plays an essential role in clarifying teachers’ role positioning and strengthening role identity.

Cognitive development theory posits that the construction of role identity, which is dynamic and complex, has two core mechanisms: “assimilation” and “adaptation.” Assimilation occurs when an individual teacher integrates newly acquired information, ideas, or knowledge from the external environment into their existing cognitive system, thus facilitating the internal integration and internalization of this information. Adaptation refers to the process in which teachers actively adjust or reconstruct their cognitive structures to better accommodate changes and challenges in the external environment when external information cannot be directly integrated into their existing frameworks. These two processes not only promote the deep absorption and internalization of external information by teachers but also elicit positive psychological feedback and behavioral responses, both of which are crucial to deepening role identity (Piaget, 1980).

In the field of SoTL, university teachers’ deep recognition and acceptance of the significance of their scholarly role serve as the internal drivers for the generation and continuous enhancement of SoTL performance. A clear interpretation of role meaning and the effective communication of information by organizational leaders play a decisive role in shaping and reinforcing teachers’ role identity (Giola and Chittipeddi, 1991). Thus, establishing a high-quality LMX is critical. LMX not only enhances teachers’ trust in their roles and provides greater resource support (Zhu, 2014), motivating them to invest more effort in school-related activities (Hardianto and Sari, 2021), but also stimulates teachers to revise their educational concepts, refine teaching methods, and invigorate academic vitality, thus strengthening their role identity (Lu, 2021). As teachers’ role identity continuously strengthens, a positive feedback loop often emerges: stronger role identity leads to more optimistic role expectations and higher performance goal-setting (Liao et al., 2010), which, in turn, encourages teachers to commit to the practice of teaching scholarship, pursue innovative approaches, and ultimately enhance their SoTL performance (Wehby-Malicki et al., 2025). Based on the preceding analysis, the following hypothesis is proposed:

*Hypothesis 1*: The quality of LMX of university teachers has a positive predictive effect on their SoTL performance. Specifically, high-quality LMX can significantly improve the SoTL performance of university teachers.

### 2.2 The mediating effect of psychological capital

In grassroots teaching organizations at universities, LMX between teachers and organizational leaders not only directly influences SoTL performance but may also have an indirect effect on psychological capital. Psychological capital, which encompasses key elements such as self-efficacy, hope, optimism, and resilience, refers to a set of psychological traits that can be developed and quantified. It is a crucial resource that fosters positive organizational behavior (Luthans et al., 2005). For university teachers, these positive psychological traits are influenced by the quality of LMX and significantly contribute to improving SoTL performance.

According to social exchange theory, high-quality LMX establishes an interactive framework based on trust and support (Martin et al., 2016; Matta et al., 2015; Rockstuhl et al., 2012). When teachers perceive trust and resource support from organizational leaders, the positive emotional bond stimulates their sense of reciprocity, motivating them to engage in their educational roles with a more positive attitude (Zhu, 2014). Specifically, this positive feedback cycle can enhance teachers’ self-efficacy, facilitate optimism, and strengthen their belief in education, thereby contributing to the accumulation and enhancement of psychological capital (Zhu, 2013).

Furthermore, according to the conservation of resource theory, individuals naturally strive to acquire and preserve valuable resources. Those with more initial resources are better positioned to acquire further resources, thus creating a spiral of resource accumulation that promotes self-development (Hobfoll et al., 2018). Psychological capital, as a scarce yet crucial resource in education (Qiu and Ye, 2023), helps teachers overcome the challenges and pressures in implementing innovation in teaching (Rharbi and Uysal, 2024), peer communication, and performance display (Bastian and Widodo, 2024). It enhances professional identity and job satisfaction, stimulates intrinsic motivation (Zhang et al., 2024), and encourages greater investment in education, teaching, and talent development (Zhang et al., 2021), thereby facilitating the “spiral gain” in SoTL performance. Based on the preceding analysis, the following hypothesis is proposed:

*Hypothesis 2*: The psychological capital of university teachers has a significant mediating effect between leader–member exchange and SoTL performance.

### 2.3 The moderating effect of leader–member exchange differentiation and traditionality

The influence of LMX on psychological capital is multifaceted and dynamic. This process is constrained by the complexity of the organizational context (Zhang et al., 2023), particularly the presence of leader–member exchange differentiation (LMX differentiation). LMX refers to the interpersonal interaction between teachers and organizational leaders at the individual level. At the organizational level, the impact of this interaction is referred to as LMX differentiation (Jia and Liden, 2013). The relationship between LMX and LMX differentiation is complex, and there is a significant interaction, which plays a crucial role in shaping psychological capital (Zhao, 2020).

Previous studies have shown that teachers’ role identity and orientation are often shaped through social comparisons with colleagues within the same grassroots teaching organization. Teachers within the same organization share similar roles and work content. This makes them more likely to engage in social comparisons (Wood, 1996). Differences in exchange relationships between leaders and subordinates provide crucial information for these comparisons (Jia and Liden, 2013). In organizations characterized by significant LMX differentiation, leaders typically place higher expectations on teachers who benefit from high-quality LMX. This positive feedback not only enhances teachers’ self-cognition (Ng, 2017) but also fosters the endogenous development of psychological capital (Saputri and Prihatsanti, 2018). Conversely, in organizations with lower LMX differentiation, the impact of LMX on psychological capital may be diminished, and as leaders convey similar expectations to all the teachers, the potential for effective comparison will be reduced. Therefore, this study hypothesizes that LMX differentiation may moderate the relationship between LMX and psychological capital.

However, the effect of LMX differentiation does not operate in isolation. It must be considered within a broader cultural context (Zhao, 2020). In particular, the influence of traditionality should not be overlooked. Traditionality primarily refers to an individual’s cognitive attitude, ideology, value orientation, temperament, and behavioral inclination toward traditional Chinese culture (Farh et al., 1997). It is the psychological component that most accurately reflects the character and value orientation of traditional Chinese individuals, which exerts a significant influence on contemporary Chinese people, particularly college teachers (Yang and Huang, 1989). Teachers with high traditionality, deeply influenced by traditional culture, are more likely to act in accordance with role responsibility, demonstrating stronger professional loyalty and moral self-discipline. In particular, their psychology and behavior tend to be more stable and less influenced by external evaluative feedback. Regardless of whether the organizational leader treats them as “fair” or not, they consistently adhere to the responsibilities and obligations inherent in their role as educators (Hui et al., 2004). Conversely, teachers with low traditionality are more likely to be driven by personal gains and losses, as well as perceptions of peer relationships. Being more sensitive to LMX differentiation, they may exhibit more volatile psychological reactions and behavioral performance. In social exchanges with organizational leaders, perceiving favorable treatment stimulates their positive psychological and behavioral responses, while perceiving unfavorable treatment may generate negative emotions, especially the potential efforts to change the status quo through “tit-for-tat” behavior (Wang Y. Q. et al., 2012).

Based on the above analysis, it is evident that the traditionality of individual teachers may create a cross-level, third-order moderating effect by re-regulating the moderating effect of LMX differentiation at the organizational level. Therefore, this study hypothesizes the following:

*Hypothesis 3*: In the process through which LMX influences psychological capital, LMX differentiation and traditionality exert a cross-level, third-order moderating effect. Specifically, when LMX differentiation is high and teachers’ traditionality is low, individuals’ sensitivity and responsiveness to leadership behavior are heightened, maximizing the impact of LMX on psychological capital.

**Figure 1:**
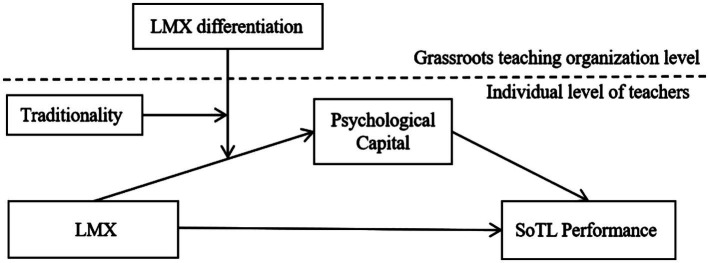
Hypothesized research model. LMX, leader–member exchange; LMX differentiation, leader–member exchange differentiation; SoTL performance, scholarship of teaching and learning performance.

In summary, this study constructs a cross-level, third-order moderating mediation model centered on individual teachers (see Figure 1). Specifically, within grassroots teaching organizations in universities, LMX influences SoTL performance through the mediating role of psychological capital. LMX differentiation within the organization and individual teachers’ traditionality exert a third-order moderating effect on the initial segment of this process. Specifically, when LMX differentiation is high within the organization and teachers’ traditionality is low, the impact of LMX on SoTL performance through psychological capital is maximized.

## 3 Materials and methods

### 3.1 Sample

The sample for this study was drawn from grassroots teaching organizations across three universities in two provinces of central China. After securing support through relationship-based resources, paired data were collected from two groups—“leaders” and “subordinates”—using convenience sampling, with a combination of online and offline methods. A total of 413 matched questionnaires were collected from 68 grassroots teaching organizations. After excluding questionnaires that did not meet the inclusion criteria, 351 valid responses from 63 organizations were retained, and an effective response rate of 84.99% was yielded. Among the respondents, 191 were men and 160 were women. The age distribution was as follows: 78.35% were under 45 years old, with 46.44% under 35 years old, 35.61% between 36 and 45 years old, and 17.95% over 46 years old. In terms of academic qualifications, 63.53% had obtained doctoral degrees, while 29.34% had master’s degrees. Regarding professional titles, 41.03% held intermediate titles, 44.44% had deputy senior titles, and 14.53% were in senior title roles.

### 3.2 Measures

This study employed established scales to construct two questionnaires. Questionnaire A included three scales: LMX, psychological capital, and traditionality, and gathered data based on teachers’ self-reports.

Questionnaire B was designed to assess SoTL performance, with data collected from evaluations by the heads of the teaching organizations. To ensure the questionnaires aligned with the language preferences and understanding of Chinese university teachers, we conducted pre-tests with the heads of grassroots teaching organizations and full-time teachers from the sample universities. Based on their feedback, we made necessary revisions to the wording of certain items. All measures were rated on a five-point Likert-type scale, where higher scores indicated greater intensity or level of the corresponding variable.

### 3.3 Research tools

#### 3.3.1 LMX

Leader–member exchange was assessed using seven items from Leader–Member Exchange 7 Questionnaire (LMX-7), a single-dimensional scale developed by Graen and Uhlbien (1995). Example items were “I understand his/her satisfaction with my professional work” and “He/She understands the difficulties and needs in my professional work.” The *α* coefficients for the scale were 0.86.

#### 3.3.2 Psychological capital

The study assessed psychological capital using 24 items from Psychological Capital Questionnaire (PCQ) (Luthans et al., 2004). It is based on the four-dimensional scale. Example items were: “I always have a firm goal and full of energy in my enthusiasm for education and adherence to the style of teaching” and “When I encounter setbacks in my work, I always recover quickly and move on.” The *α* coefficients for the scale were 0.925.

#### 3.3.3 Traditionality

Traditionality was measured by using a five-dimensional scale from Multidimensional Scale of Chinese Individual Traditionality (MS-CIT), which was developed by Yang and Huang (1989) and was simplified to suit the context of university teachers. Three dimensions were selected—respect for authority, safety, and self-preservation—comprising a total of six items. Example items include: “Obedience to authority and respect for the virtues that children should learn” and “As long as people abide by government decrees, they do not need to seek further understanding of the truth.” The Cronbach’s *α* for the traditionality scale was 0.744.

#### 3.3.4 SoTL performance

SoTL performance was measured by using a two-dimensional scale from Job Performance Scale, which was developed by Vanscotter and Motowidlo (1996), and was adjusted to better reflect the SoTL performance of university teachers. The two dimensions—task performance and contextual performance—comprised a total of seven items. Example items include: “He/she pays attention to the summary and accumulation of educational and teaching methods, develops a personal teaching style, or produces relevant teaching outcomes,” “Team colleagues understand and recognize his/her teaching philosophy,” and “He/she maintains good relationships with colleagues and often assists them in completing their study.” The Cronbach’s *α* for the SoTL performance scale was 0.851.

#### 3.3.5 LMX differentiation

LMX differentiation was measured by following the approach proposed by Leblanc and González-Romá (2012). After assessing the individual LMX levels of teachers, the standard deviation of LMX scores was calculated within groups (organized by grassroots teaching organizations) to determine the degree of LMX differentiation at the team level. A higher standard deviation indicates greater LMX differentiation within the grassroots teaching organization. The Cronbach’s *α* for the LMX differentiation scale in this study was 0.862.

### 3.4 Analysis method

In this study, all variables were first standardized. Descriptive statistics and correlation analyses were conducted by using SPSS 23.0. After controlling for age and professional title, the PROCESS macro developed by Hayes (2013) was employed to analyze the moderating and mediating effects. Subsequent tests revealed that the variance inflation factors (VIFs) for all variables did not exceed 2.9. This indicates that multicollinearity was not a concern.

## 4 Results

### 4.1 Common method variance

In accordance with Zhou and Long (2004), this study employed both program and statistical controls to reduce and test for common method variance (CMV). Procedurally, following Podsakoff et al. (2003), the research data were collected in three stages from two groups of “leaders” and “subordinates.” The first stage involved a preliminary investigation of the organizational environment and the establishment of a numbering system. Prior to the formal investigation, the researcher coordinated with the heads of the grassroots teaching organizations to assign numbers to the organizations and teachers. Participants were informed appropriately, ensuring that only the numbers appeared on the questionnaires—no personal identifying information was collected. To ensure that all results were kept confidential, these numbers were used solely for data pairing and the research team adhered strictly to research ethics. The second stage involved surveying teachers within the respective organizations, who were asked to evaluate their leader–member exchange, psychological capital, and traditionality levels. The third stage was conducted two months after the completion of the second stage, where the heads of the organizations were asked to evaluate the teachers’ SoTL performance within the grassroots teaching organizations. Statistically, Harman’s single-factor test was applied, revealing that seven factors with eigenvalues greater than 1 emerged. The first common factor accounted for 34.957% of the total variance, which is below the 50% threshold commonly used in organizational behavior research. This suggests that common method variance was not a significant issue in the data of this study (Hair et al., 1998).

### 4.2 Descriptive statistics and correlations

Table 1 presents the descriptive statistics and correlation analysis results of this study. Specifically, the correlation between LMX and SoTL performance is the strongest (*r* = 0.742), indicating a strong positive relationship. The correlation coefficient between psychological capital and SoTL performance is also relatively high (*r* = 0.677), suggesting that both factors exert a strong positive influence on SoTL performance. Traditionality is also positively correlated with SoTL performance (*r* = 0.584), implying that, within the context of Chinese culture, traditionality continues to play a significant role in enhancing SoTL performance. The correlation coefficient between LMX differentiation and SoTL performance is slightly lower (*r* = −0.229), suggesting that LMX differentiation exerts a small negative effect on SoTL performance. The pairwise correlations among leader–member exchange, psychological capital, and SoTL performance provide the foundation for the subsequent mediation effect analysis.

**Table 1:** Descriptive statistics and correlation analysis of variables.

Variable	M	SD	1	2	3	4	5
1. LMX	3.956	0.676	—				
2. Psychological capital	4.013	0.567	0.779^***^	—			
3. Traditionality	2.803	0.725	0.582^***^	0.609^***^	—		
4. LMX differentiation	0.565	0.224	−0.225^***^	−0.205^***^	−0.178^**^	—	
5. SoTL performance	3.869	0.619	0.742^***^	0.677^***^	0.584^***^	−0.229^***^	—

*N* = 351; ***p < 0.001; **p < 0.01; M, Mean; SD, Standard Deviation; LMX, leader-member exchange; SoTL, scholarship of teaching and learning.

### 4.3 Testing of mediation effects

In accordance with the guidelines of Hayes (2013) and Wen and Ye (2014), this study tested the moderated mediation model in two stages: first, testing the mediation effect, and second, assessing the moderating mediation effect.

To explore the mediating role of psychological capital between leader–member exchange and SoTL performance, the study employed SPSS PROCESS plug-in (Model 4) for regression analysis. As shown in Table 2, leader–member exchange significantly and positively predicted psychological capital (a = 0.766, *p* < 0.001), suggesting that a high-quality leader–member exchange relationship can enhance teachers’ psychological capital. When both leader–member exchange and psychological capital were included in the regression equation, both factors have a significant positive impact on SoTL performance (c’ = 0.539, *p* < 0.001; b = 0.258, *p* < 0.001). This confirms the partial mediating role of psychological capital. Furthermore, when using the variance-corrected non-parametric bootstrap method (5,000 samples), it was found that psychological capital significantly mediated the relationship between leader–member exchange and SoTL performance (ab = 0.198, Boot SE = 0.048, 95% CI = [0.105, 0.290]). The mediation effect, which accounted for 26.866% of the total effect, underscores the critical role of psychological capital in transmitting the effects of leader–member exchange on SoTL performance.

**Table 2:** Regression results for the main effect and the mediation effect.

Variable	Equation 1 (dependent: Psychological capital)			Equation 2 (dependent: SoTL performance)		
	*β*	*SE*	*t*	*β*	*SE*	*t*
LMX	0.766 ^***^	0.034	22.79	0.539 ^***^	0.061	8.829
Psychological capital				0.258 ^***^	0.064	4.05
Age	−0.01	0.045	−0.232	0.074	0.044	1.704
Professional title	0.167^*^	0.062	2.698	−0.099	0.071	−1.391
*R* ^2^	0.618			0.579		
*F*	102.129 ^***^			109.167 ^***^		

*N* = 315; ***p < 0.001; **p < 0.01; *p < 0.05; LMX, leader-member exchange; SoTL, scholarship of teaching and learning; Bootstrap sample size = 5000.

### 4.4 Testing of cross-level moderating mediating effects

According to the suggestion of Hayes (2013), this study used Model 11 of PROCESS plug-in to further explore how leader–member exchange differentiation, traditionality, and their interaction with leader–member exchange regulate the above mediating paths across levels. According to the method requirements, this study analyzed two regression equations.

Equation 1 tested the direct impact of leader–member exchange on psychological capital and examined the predictive power of the interaction term involving leader–member exchange differentiation, traditionality, and leader–member exchange on psychological capital. The results, presented in Table 3, not only indicate that leader–member exchange significantly predicts psychological capital, but also show the significance of the third-order interaction of leader–member exchange, leader–member exchange differentiation, and traditionality. This suggests that the combined effect of these three factors exerts a cross-level moderating effect on psychological capital. Consequently, the effect observed in Equation 1 is statistically significant.

**Table 3:** Results of analysis cross-level moderated mediation model analysis.

Predictors	Psychological capital			SoTL performance		
	*β*	*SE*	*t*	*β*	*SE*	*t*
LMX	0.541^***^	0.048	11.23	0.539^***^	0.061	8.829
LMX differentiation	0.062	0.037	1.668			
LMX × LMX differentiation	0.008	0.045	0.165			
Traditionality	0.253^***^	0.04	5.844			
LMX × Traditionality	−0.113^**^	0.042	−2.681			
LMX differentiation × Traditionality	0.017	0.041	0.409			
LMX × LMX differentiation × Traditionality	−0.064^*^	0.028	−2.287			
Psychological capital				0.258^***^	0.064	4.05
Age	0.001	0.041	0.027	0.074	0.044	1.704
Professional title	0.121^*^	0.057	2.134	−0.099	0.071	−1.391
*R^2^*	0.689			0.579		
*F*	71.794^***^			109.167^***^		

*N* = 351; ^***^p < 0.001; ^**^p < 0.01; ^*^p < 0.05; LMX, leader-member exchange; SoTL, Scholarship of teaching and learning.

Equation 2 examines the direct impact of leader–member exchange and psychological capital on the SoTL performance and verifies the second half of the moderating mediating effect. The results show that both leader–member exchange and psychological capital have a significant positive impact on the SoTL performance. This confirms that the second half of the moderating mediating effect is also effective, and the effect of Equation 2 is significant.

In summary, in the grassroots teaching organizations of universities, the leader–member exchange differentiation and teacher traditionality not only independently affect psychological capital but also jointly moderate the first half of the intermediary path of “leader-member exchange → psychological capital → SoTL performance” through a cross-level, third-order interaction.

**Figure 2:**
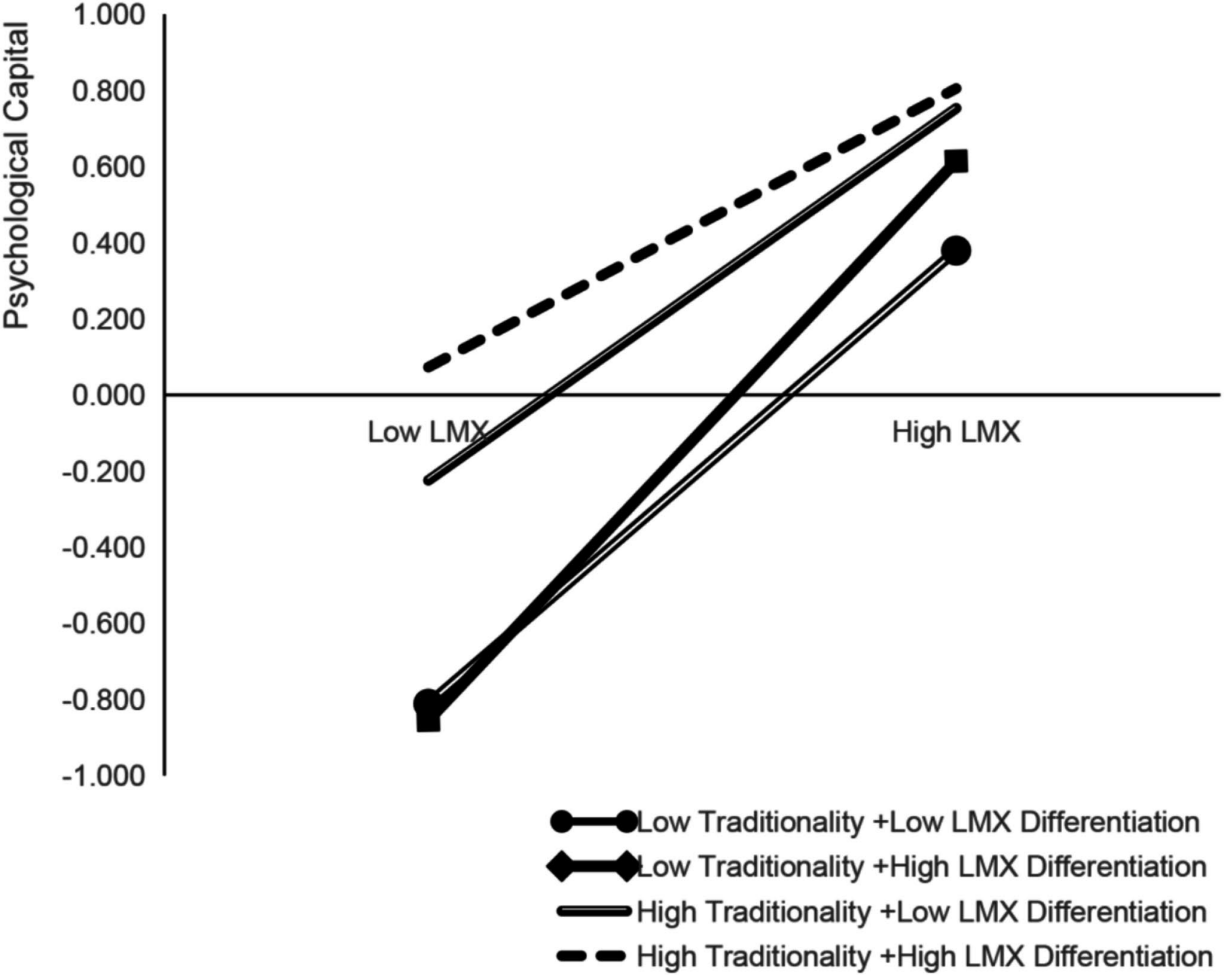
Diagram illustrating the third-order moderating effect. LMX, leader-member exchange.

To further explore the moderating role of leader–member exchange differentiation and traditionality in the influence path, this study used SPSS statistical software. Based on the standard of one standard deviation above and below the mean, the two variables were divided into high and low groups, respectively, and then classified into four groups for simple slope analysis (see Figure 2). The results show that the moderating effects in each group are significant, but their intensity and patterns differ notably. Specifically, when teachers’ traditionality is high, the impact of leader–member exchange quality on psychological capital remains relatively stable. In organizational contexts with high leader–member exchange differentiation, its positive effect on psychological capital is weaker, whereas in environments with lower differentiation, this positive effect is stronger. Conversely, for teachers with low traditionality, the quality of leader–member exchange has a more pronounced impact on psychological capital. Particularly in contexts with significant leader–member exchange differentiation, this effect reaches its peak. This highlights that low-traditionality teachers are especially sensitive to the quality of leader–member exchange in highly differentiated environments.

On the whole, this study verifies that the leader–member exchange of university teachers acts on the SoTL performance through psychological capital, which is a complex mechanism moderated by the leader–member exchange differentiation at the organizational level and the traditionality at the individual level of teachers. As shown in Table 4, for low-traditionality teachers, the indirect effect of leader–member exchange on the SoTL performance through psychological capital is the most significant in the organizational context with high leader–member exchange differentiation; on the contrary, for high-traditionality teachers, the intensity of this indirect effect is minimized in the context of leader–member exchange differentiation and the difference in the standardized effect coefficient between the two reaches 0.091. This further confirms the robustness and explanatory power of the cross-level third-order moderating mediation model.

**Table 4:** Bootstrap analysis of significance test of mediation effect.

Type	Moderator variables		Effect value	Bootstrap SE	Bootstrap CI	
					Lower	Upper
LMX → Psychological Capital→ SoTL Performance	High Traditionality	High LMX Differentiation	0.096	0.028	0.015	0.162
		Low LMX Differentiation	0.125	0.037	0.065	0.209
	Low Traditionality	Low LMX Differentiation	0.15	0.041	0.083	0.246
		High LMX Differentiation	0.187	0.045	0.104	0.282

LMX, leader-member exchange; SoTL, scholarship of teaching and learning.

## 5 Discussion

Strengthening organizational relationships is foundational to building an efficient grassroots teaching organization. In the field of higher education, grassroots teaching organizations within colleges and universities serve not only as centers for knowledge dissemination and innovation but also as essential platforms for teamwork and personal growth among teachers. Therefore, fostering harmonious relationships between organizational leaders and members is a critical factor in developing the institution’s connotation and academic excellence. This study explores the multifaceted impact of social exchange behavior and emphasizes its role in building teachers’ psychological capital and enhancing SoTL performance. The discussion aims to elaborate on the study’s findings and significance, particularly focusing on the roles of LMX, psychological capital, LMX differentiation, and traditionality in shaping SoTL performance.

### 5.1 The role of LMX in improving SoTL performance

LMX is a crucial factor affecting the SoTL performance of university teachers. High-quality LMX provides teachers with more resources and support, which in turn strengthens their role identity and professional positioning. The findings of this study align with previous research in organizational settings, which has shown that in enterprise organizations, LMX positively influences job performance (Zhang et al., 2020; Wang Z. et al., 2012; Qi et al., 2023; Rockstuhl et al., 2012; Tang and Vandenberghe, 2021). In educational settings, teacher–leader LMX also significantly enhances job performance (Mahinay, 2024; Hasyyati et al., 2022; Tengah and Zumrah, 2023; Çevik, 2025). This study further illustrates that teachers’ LMX has a positive impact on job satisfaction and SoTL performance within Chinese universities. When teachers perceive strong support from their leaders, they are more likely to engage in innovative teaching practices, which will further improve their SoTL performance.

### 5.2 The mediating role of psychological capital

Psychological capital plays a vital role in improving SoTL performance and acts as an important mediator between LMX and SoTL performance. Psychological capital encompasses self-efficacy, hope, optimism, and resilience. When college teachers experience high-quality LMX, their psychological capital is enhanced. This finding is consistent with extensive research on psychological capital in academic literature, which has verified its role in improving job performance and organizational citizenship behavior (Xu et al., 2022; Avey et al., 2011; Nolzen, 2018; Peterson et al., 2011; Yildiz, 2019). These studies have also highlighted how psychological capital mediates the relationship between LMX and job performance (Liao et al., 2017; Upadhyay and Kumar, 2020). The data from this study show that the mediating effect of psychological capital accounts for 26.866% of the total effect between LMX and SoTL performance. As psychological capital improves, SoTL performance is significantly enhanced. This underscores the importance of psychological capital in transferring the positive effects of LMX to SoTL performance, suggesting that fostering psychological capital may be a key strategy to enhance teachers’ wellbeing and promote higher SoTL performance.

### 5.3 The moderating effect of LMX differentiation and traditionality

This study reveals that LMX differentiation and traditionality play a complex moderating role in the relationship between LMX and psychological capital. LMX differentiation refers to the variation in the quality of relationships between leaders and different members, which can either amplify or weaken the impact of LMX on psychological capital. In environments with high LMX differentiation, teachers with high-quality LMX may receive more support and have higher expectations placed upon them, and thus their psychological capital will be enhanced. However, this effect is influenced by the teachers’ level of traditionality. Specifically, for individuals with low traditionality, in a context of high LMX differentiation, LMX has a particularly significant impact on both psychological capital and SoTL performance. Although LMX differentiation generally leads to a stronger effect on psychological capital, personal traditionality, which can influence how teachers interpret leadership behavior, will in turn produce different outcomes. Personal traditionality reflects an individual’s alignment with leadership and affects their interpretation of leadership behavior. For low-traditionality teachers, if they perceive a weak LMX in a highly differentiated LMX environment, they may interpret leadership behavior negatively, believing leaders are intentionally distancing themselves. This exacerbates the loss of psychological capital. Conversely, highly traditional teachers tend to understand leadership decisions more positively and offer reasonable explanations for leaders’ behavior, such as attributing it to the broader considerations of leadership or prioritizing the needs of other employees. As a result, their psychological capital is less impacted.

The positive indirect effect of personal tradition identified in this study aligns with previous research (Hu et al., 2020; Wan et al., 2022; Zheng and Ahmed, 2024), which has shown that personal traditionality can enhance the positive influence of LMX on job crafting. Specifically, LMX promotes job crafting only for employees with higher personal traditionality, whereas it has no significant impact on employees with lower traditionality. This study extends this finding from enterprise settings to the university context, thus offering new perspectives for future research. Future studies could explore whether personal traditionality can mitigate the effects of other negative environmental factors on university teachers. For example, researchers could examine whether highly traditional teachers are less affected by the negative impact of abusive leadership.

It is worth noting that while a small number of studies have focused on the moderating variables of LMX, only a few have explored the moderating effects of individual-level and organizational-level factors on LMX. For instance, Erdogan and Enders (2007) found that managers’ perceived organizational support moderates the relationship among LMX, job satisfaction, and performance. When managers perceive higher organizational support, the positive impact of LMX on subordinates’ job satisfaction and performance is stronger. Ali et al. (2024) discovered that AI tools moderate the effect of LMX on team performance and psychological empowerment. When AI tools are effectively used, the LMX relationship has a stronger impact on team performance. Chiu et al. (2022) found that psychological contract breach negatively moderates the relationship between LMX and psychological empowerment. When psychological contract breach is low, LMX has a greater impact on psychological empowerment. Tran et al. (2020) found that perceived organizational support moderates the relationship between LMX and social capital, with higher perceived support strengthening the impact of LMX on social capital. By comprehensively examining the moderating effect of traditionality and LMX differentiation, this study fills a gap in the research field and contributes to the further development of LMX theory.

### 5.4 Practical implications

By highlighting the influence mechanism of LMX on the SoTL performance of university teachers, this study reveals the mediating role of psychological capital and the moderating effects of LMX differentiation and traditionality. These effects vary across different organizational environments and among teachers with varying levels of traditionality. Therefore, to enhance SoTL performance and promote the high-quality development of higher education in Chinese universities, several organizational strategies are recommended.

First, optimize the quality of LMX relationships. High-quality LMX is essential to improve the SoTL performance of university teachers. Universities should prioritize leadership training to enhance leaders’ ability to build trust, provide support, and set clear expectations. Team-building activities should be used to foster cooperation and emotional communication between teachers to create a positive and harmonious working environment. Furthermore, decentralizing management authority, providing grassroots teaching organizations with more resources and autonomy, and supporting the development of academic activities will improve the quality of LMX.

Second, develop psychological capital. Psychological capital plays a crucial role as an intermediary factor in improving SoTL performance. Therefore, universities should develop strategies to enhance teachers’ psychological capital. This can be achieved through mentorship programs that help teachers establish professional beliefs and enhance their sense of mission. By optimizing the teaching evaluation system, universities can better align teaching effectiveness with student growth, thereby enhancing teachers’ professional pride. Additionally, mental health education should be strengthened to improve psychological qualities that can reduce work pressure and improve job satisfaction, such as optimism, resilience, and hope.

Third, leverage the positive role of traditionality. This study finds that traditionality can positively affect SoTL performance and buffer the negative effects of low LMX on psychological capital. Compared with teachers with low traditionality, teachers with high traditionality have relatively higher psychological capital even when the quality of leader–member exchange is low (Figure 2). Despite the criticisms of traditionality in the context of China’s higher education, the findings suggest that we should recognize its positive aspects. Universities can guide teachers in revisiting Chinese cultural classics and exploring the beneficial connotations of traditionality in modern higher education. While emphasizing traditionality, universities should also foster a professional environment conducive to innovation and creativity to encourage teachers to reflect and adapt to the needs of modern education. This integration of traditionality and modernity can drive SoTL performance.

Fourth, control the negative impact of LMX differentiation. Excessive differentiation in LMX can create feelings of injustice and undermine team cohesion. Universities should work on improving leaders’ fairness awareness and management skills to establish a fair resource allocation system and minimize perceptions of favoritism. Leaders should aim to establish high-quality LMX with key members and apply appropriate differentiated management to mitigate the negative impacts of LMX differentiation.

Fifth, target intervention for teachers with low traditionality and high LMX differentiation. For teachers with low traditionality and high LMX differentiation, LMX has the most significant impact on psychological capital and SoTL performance (Figure 2). To offer attention and support, universities should identify these teachers and actively engage with them to improve their LMX quality. Additionally, universities can provide social training skills to enhance communication and encourage these teachers to actively interact with leaders and seek help, ultimately improving their LMX.

## 6 Limitations and future research

This study employs a cross-sectional design, which presents limitations regarding causal inferences and makes it challenging to fully elucidate the dynamic relationships between variables. Furthermore, the research sample is confined to 68 grassroots teaching organizations from three universities across two provinces in central China, limiting the generalizability and representativeness of the findings. Given these limitations, future studies should consider employing a longitudinal design to investigate the long-term dynamic relationships between the variables in greater depth. Simultaneously, the sample scope should be broadened to encompass additional regions and diverse types of universities, thereby enhancing the generalizability and robustness of the research conclusions. Additionally, using modern technologies such as big data and artificial intelligence could facilitate further exploration of moderating factors within various cultural contexts and educational environments, identify additional influencing variables, and assess the applicability and generalizability of the study’s findings.

## 7 Conclusion

This study confirms the significant impact of LMX on SoTL performance. It reveals the mediating role of psychological capital and the moderating effects of LMX differentiation and personal traditionality. The results indicate that university faculty with high-quality LMX tend to demonstrate higher levels of SoTL performance. High-quality LMX can foster a positive mechanism in which faculty members perceive trust and support from organizational leaders, leading to a more positive psychological state that subsequently enhances teaching effectiveness. Additionally, the study identifies that LMX differentiation within grassroots teaching organizations and individual traditionality moderate the effect of LMX on psychological capital. Specifically, when faculty members exhibit low personal traditionality and operate within high LMX differentiation environments, the influence of LMX on both psychological capital and SoTL performance is more pronounced. This suggests that traditionality can serve as a secondary moderator of the moderating role of LMX differentiation. These findings underscore the profound significance of organizational relationship-building in promoting the teaching development of university faculty. As the first study to integrate LMX differentiation, traditionality, and psychological capital to systematically examine the mechanism by which LMX affects teaching and academic performance, this research offers new insights and theoretical advancements for the application of LMX theory in higher education.

## Data Availability

The raw data supporting the conclusions of this article will be made available by the authors, without undue reservation.
